# Projected near-future CO_2_ levels increase activity and alter defensive behaviours in the tropical squid *Idiosepius pygmaeus*

**DOI:** 10.1242/bio.20149894

**Published:** 2014-10-17

**Authors:** Blake L. Spady, Sue-Ann Watson, Tory J. Chase, Philip L. Munday

**Affiliations:** 1 College of Marine and Environmental Sciences, James Cook University, Townsville, QLD 4811, Australia; 2 ARC Centre of Excellence for Coral Reef Studies, James Cook University, Townsville, QLD 4811, Australia

**Keywords:** Ocean acidification, Cephalopod, Anti-predator behaviour, Escape, Avoidance, Startle response

## Abstract

Carbon dioxide (CO_2_) levels projected to occur in the oceans by the end of this century cause a range of behavioural effects in fish, but whether other highly active marine organisms, such as cephalopods, are similarly affected is unknown. We tested the effects of projected future CO_2_ levels (626 and 956 µatm) on the behaviour of male two-toned pygmy squid, *Idiosepius pygmaeus*. Exposure to elevated CO_2_ increased the number of active individuals by 19–25% and increased movement (number of line-crosses) by nearly 3 times compared to squid at present-day CO_2_. Squid vigilance and defensive behaviours were also altered by elevated CO_2_ with >80% of individuals choosing jet escape responses over defensive arm postures in response to a visual startle stimulus, compared with 50% choosing jet escape responses at control CO_2_. In addition, more escape responses were chosen over threat behaviours in body pattern displays at elevated CO_2_ and individuals were more than twice as likely to use ink as a defence strategy at 956 µatm CO_2_, compared with controls. Increased activity could lead to adverse effects on energy budgets as well as increasing visibility to predators. A tendency to respond to a stimulus with escape behaviours could increase survival, but may also be energetically costly and could potentially lead to more chases by predators compared with individuals that use defensive postures. These results demonstrate that projected future ocean acidification affects the behaviours of a tropical squid species.

## INTRODUCTION

Over the last 250 years, anthropogenic fossil fuel combustion and deforestation have increased atmospheric carbon dioxide (CO_2_) concentrations by almost 40% from 280 ppm to over 395 ppm (Collins et al., 2013). This increase has occurred at least an order of magnitude faster than at any time in the past million years (Doney and Schimel, 2007). Nearly one third of the anthropogenic CO_2_ released into the atmosphere is absorbed by the oceans (Sabine and Feely, 2007) and this uptake of CO_2_ alters ocean chemistry causing a reduction in seawater pH, a process referred to as ocean acidification (Doney et al., 2009). The partial pressure of CO_2_ (*p*CO_2_) in the ocean also increases as atmospheric CO_2_ levels rise, because the atmosphere and surface ocean are in approximate gas equilibrium (Doney, 2010). On the current emissions trajectory, atmospheric CO_2_ is projected to exceed 900 ppm by 2100 and ocean pH will drop by a further 0.3–0.4 units compared with current-day values (Collins et al., 2013). Such dramatic changes to ocean chemistry could affect many marine organisms with potentially far-reaching impacts on marine ecosystems (Hoegh-Guldberg et al., 2007; Fabry et al., 2008; Doney et al., 2009).

Reduced seawater pH and elevated CO_2_ can have a range of effects on marine organisms, including reduced calcification rates (Gazeau et al., 2007; Kleypas and Yates, 2009; Orr et al., 2009), altered metabolic rates (Pörtner et al., 2004; Gutowska et al., 2008; Rosa and Seibel, 2008), and reduced rates of growth, development and survival (Fabry et al., 2008; Kurihara et al., 2008; Doney, 2010). Additionally, elevated CO_2_ alters behaviour and impairs decision-making processes in some marine animals (Briffa et al., 2012). In marine fishes, exposure to CO_2_ levels projected to occur in the ocean by the end of the century alters olfactory (Munday et al., 2009; Dixson et al., 2010) and auditory preferences (Simpson et al., 2011) and impairs visual function (Ferrari et al., 2012; Chung et al., 2014). These changes to sensory systems in turn lead to changes in behaviour. Elevated CO_2_ also increases activity levels and increases boldness in reef fishes (Munday et al., 2010). As a result of this altered behaviour, mortality rates from predation are significantly higher in juvenile fishes exposed to elevated CO_2_ compared with controls (Munday et al., 2010; Ferrari et al., 2011). The cause of these diverse sensory and behavioural effects in fish appears to be interference with the function of type A *y*-aminobutyric acid neurotransmitter (GABA_A_) receptors as a result of the compensatory changes in transmembrane bicarbonate (HCO_3_^2−^) and chloride (Cl^−^) ion concentrations that occur during acid–base regulation in a high CO_2_ environment (Nilsson et al., 2012; Hamilton et al., 2014; Chivers et al., 2014).

While most research into the behavioural effects of elevated CO_2_ has been conducted on fish, exposure to high CO_2_ can also affect the behaviour of some marine invertebrates (Briffa et al., 2012). For example, recent studies show that CO_2_ levels projected for the end of the century affect the response of gastropod molluscs to predator cues. The self-righting times of *Concholepas concholepas* are shortened by near-future CO_2_ levels (716 and 1,036 µatm) both in the presence and absence of a predator (Manríquez et al., 2013). The predator avoidance behaviour of *C. concholepas* is also affected by elevated CO_2_ (1,000 µatm), with reduced avoidance of predator odours in a Y-maze (Manríquez et al., 2014). In the jumping conch snail (*Gibberulus gibberulus gibbosus*), elevated CO_2_ (961 µatm) impairs predator–escape behaviour, reducing the proportion of snails that jump from a predator, and increasing latency to jump as well as altering the escape trajectories of snails that do jump to escape a predator (Watson et al., 2014). Furthermore, the alteration of escape responses in gastropods at elevated CO_2_ may be caused by interference of GABA_A_ neurotransmitter receptor function (Watson et al., 2014), just as in fish (Nilsson et al., 2012). The similarity in the apparent mechanism of behavioural impairment in fishes and gastropod molluscs, and the widespread occurrence of GABA_A_ neurotransmitter receptors throughout the animal kingdom, suggests that elevated CO_2_ could potentially affect the behaviour of many marine organisms.

Cephalopods are marine invertebrates with elaborate sense organs, large brains, and complex behaviours comparable to those of fishes (Hanlon and Messenger, 1996). A number of studies have examined the effects of elevated CO_2_ on the metabolic rates (Gutowska et al., 2008; Rosa and Seibel, 2008; Gutowska et al., 2010; Hu et al., 2014), development (Pecl and Jackson, 2008; Kaplan et al., 2013) and acid–base regulation (Hu et al., 2013; Hu et al., 2014) of squid and cuttlefish, but no studies have investigated the potential effects of ocean acidification on cephalopod behaviour. In squid, GABA_A_ receptors act as ionotropic neurotransmitters (neurotransmitter-gated ion channels) (Conti et al., 2013), suggesting potential similarities with fish. Most squid have a ‘giant fibre’ neural network system, which is used in the rapid backwards jet-propelled escape response triggered by a sensory input (predominately visual) (Williamson and Chrachri, 2004), similar to the C-start escape response found in many fishes (Domenici and Blake, 1997; Wöhl and Schuster, 2007). Although GABA expression in squid is relatively low compared to vertebrates (Conti et al., 2013), evidence that elevated CO_2_ alters behaviour in other mollusc species (Manríquez et al., 2013; Manríquez et al., 2014; Watson et al., 2014) suggests that predator–prey interactions and other ecologically important behaviours in squid could be vulnerable to projected end-of-century CO_2_ levels. Squid prey on a wide range of marine organisms and also form a significant part of the diet of many predator species including cetaceans, pinnipeds and birds, as well as a wide range of teleost fishes and sharks (Hanlon and Messenger, 1996). Consequently, any changes to the predator–prey dynamics of squid, such as their defensive options, could have ramifications for ecosystem structure.

Cephalopod defence can be categorized into primary defence (avoidance by using cryptic behaviours or camouflage), secondary defence (used to threaten, startle or bluff the predator) and escape responses (Hanlon and Messenger, 1996; Mather et al., 2014). Secondary defence and escape responses involve direct interaction with the predator (Hanlon and Messenger, 1996; Mather et al., 2014). The objective of secondary defence and escape responses are to break the potential predator's search image prior to or during a pursuit (Mather et al., 2014). Examples of secondary defence include ‘deimatic displays’ and certain arm postures including the ‘anchor display’ (Moynihan, 1983; Hanlon and Messenger, 1996). In contrast, escape behaviours are usually unsystematic and erratic movements or actions used to confuse or disorient the predator, such as heavy inking, flashing of patterns, and erratic jetting (Humphries and Driver, 1970; Edmunds, 1974; Hanlon and Messenger, 1996). The escape response is usually used as a last resort when a chase by the predator is imminent or already in progress, as fleeing from a non-pursuing predator may evoke a chase response (Rosier and Langkilde, 2011).

In this study, we tested the effects of two near-future CO_2_ levels (626 and 956 µatm) on activity levels and behavioural responses to a visual startle stimulus in the two-toned pygmy squid, *Idiosepius pygmaeus*, in an aquarium setting. The CO_2_ treatments were selected to match projected CO_2_ levels in the atmosphere and ocean by the end of this century on a moderate (RCP6) or high (RCP8.5) emission trajectory (Collins et al., 2013). *I. pygmaeus* is a tropical species of squid within the order *Sepiolida*, and it can be found in the Indo-Pacific from the South China Sea to northern Australia and inhabits shallow coastal waters (Semmens et al., 1995) (I. Barratt and L. Allcock, *Idiosepius pygmaeus*, IUCN 2013 red list of threatened species, http://www.iucnredlist.org/details/162604/0 (accessed 24 October 2013)). Although *I. pygmaeus* is a less studied species compared to other cephalopods, its small size and adjustability to captivity make it highly suitable for laboratory studies. Average body size of this species is 8 mm in length and individuals have a life span of about 90 days (Moynihan, 1983; Hanlon and Messenger, 1996). Due to its small size, *I. pygmaeus* relies heavily on primary defence to avoid predation and individuals are often seen floating at the surface in a head down posture using deceptive camouflage to resemble flotsam (Moynihan, 1983). *Idiosepius* also attach to sea grass or flotsam using an adhesive gland on the mantle, which is unique to the genus, to rest while blending in with the environment (von Byern and Klepal, 2006). These strategies may be important for avoiding predators as well as conserving energy. It was predicted that elevated CO_2_ may alter activity levels and response to a threat stimulus in the pygmy squid, as previously observed in fish and other mollusc species.

## MATERIALS AND METHODS

### Collection

Two-toned pygmy squid, *Idiosepius pygmaeus*, were collected during September and October 2013 from Cleveland Bay in Townsville, Queensland, Australia (19°25′S, 146°82′E). Animals were captured using a 500 µm-mesh triangular frame dip net and transported immediately to James Cook University, Townsville. Here, squid were housed in 53-litre rectangular tanks (56×38×25 cm) at a maximum of five individuals per tank, with six tanks at each of the three different CO_2_ levels. Each tank included rocks and PVC pipe structures that could be used as hiding places. *I. pygmaeus* readily adjust to captivity (Moltschaniwskyj et al., 2007) and in the current study were observed regularly feeding, copulating and laying eggs at all CO_2_ treatment levels. Squid were sexed by visual inspection of live animals. Sex was determined by observing the testes present underneath the fins in males or the nidamental glands found in the lower mantle of females. Only males were used in the experiment as preliminary trials revealed potential variation in behaviour and activity between sexes and, due to their solitary nature (Lewis and Choat, 1993), the number of females collected was insufficient to perform reliable tests. Squid were kept in treatment tanks for five days during which time they were fed *Artemia* nauplii ad libitum once per day in the morning. The food remained in the tanks in abundance throughout the day and squid were observed to regularly graze throughout the day as previously observed in captivity (Moynihan, 1983; Jackson, 1989). Food was withheld on the day of behavioural trials. A treatment period of five days was chosen as previous experiments have demonstrated that the behavioural effects of elevated CO_2_ are evident within four days of exposure in fish (Munday et al., 2010; Ferrari et al., 2012) and five days in molluscs (Watson et al., 2014), and that behaviour does not adjust following longer exposures of weeks to months (Munday et al., 2013b; Munday et al., 2014). We confirm that all experiments comply with all relevant institutional and national animal welfare laws, guidelines and policies.

### CO_2_ treatment systems

Three separate 8000 L recirculating systems were each set to a different pH level and corresponding *p*CO_2_: 1) a current-day control (447 µatm), 2) a lower end of the century projection (moderate CO_2_ 626 µatm) and, 3) an upper end-of-century projection (high CO_2_ 956 µatm) (Collins et al., 2013). A pH control system (AT Control, Aqua Medic, Germany) dosed CO_2_ into 3000 L sumps to achieve the necessary pH level for each CO_2_ treatment. pH dosing set points were adjusted as needed to maintain the desired *p*CO_2_ in each treatment. Equilibrated seawater from each system was then delivered to aquaria containing squid at a rate of 1.5 L min^−1^. pH_NBS_ and temperature were recorded every other day using a pH electrode (SevenGo Pro, Mettler Toledo, Switzerland) and a temperature probe (Comark C26, Norfolk, UK). Total alkalinity was estimated weekly by Gran Titration (888 Titrando Metrohm AG, Switzerland) using certified reference material from Dr. A.G. Dickson (Scripps Institution of Oceanography). Salinity was measured weekly using a conductivity sensor (HQ15d, Hach, Loveland, CO, USA). Carbonate chemistry parameters (Table 1) were calculated in CO2SYS (Pierrot et al., 2006) using the constants K1 from Mehrbach et al. (Mehrbach et al., 1973) refit by Dickson and Millero (Dickson and Millero, 1987) for KHSO_4_. There was some variation in salinity among treatments, but *I. pygmaeus* regularly experience substantial fluctuations in salinity in their shallow coastal habitat.

**Table 1.**
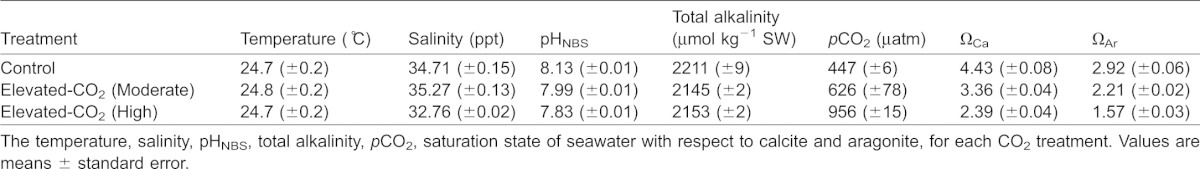
Seawater carbonate chemistry

### Activity observations

Activity levels of individual squid were recorded after five days in CO_2_ treatment. Individuals were carefully transferred from their holding tank to a clear plastic aquarium (34×20×18 cm) containing 11 litres of seawater from their CO_2_ treatment. The size of the observational tank is not expected to have a limiting effect on squid activity. During activity observations, movement was generally observed as a single short distance jet followed by a brief rest, then another short jet in a seemingly random direction. Individuals did not swim/move into the walls of the aquarium and, therefore, did not appear to be restricted in their movements. The front and left side of the aquarium contained gridded lines spaced 2 cm apart (Munday et al., 2013b). A mirror was placed at a 45° angle from the left rear corner of the aquarium to gain a front and side view of squid activity from a single viewing point. A video camera (Canon PowerShot G15) on a tripod was placed at a distance of 60 cm from the aquarium. Each individual was video-recorded for a total of 10 minutes after the observer had exited the room. No experimental manipulation was conducted during this observational period.

When analysing the video, data were recorded only from the last 5 minutes in order to allow a 5-minute acclimatisation period. During the last 5-minute period, the number of times that the subject's eye crossed one of the gridded lines on the front of the aquarium was recorded and then the same section of video was re-played to count the number of line crosses on the side of the aquarium, which was viewed through the mirror. These two line cross values were summed in order to give a measure of total movement on a 3-dimentional plane. Some squid used their adhesive gland to attach themselves to the wall of the tank during the 5-minute acclimatisation period and remained there throughout the 5-minute observation period. These individuals, with an activity level of 0 line crosses, were categorized as “at rest”. Squid with between 1 and 25 line crosses were characterized as “low activity”. These individuals remained relatively still at the surface using deceptive camouflage as often seen in the wild, only using their fins to subtly change their direction or orientation. Squid with over 25 line crosses actively jetted back and forth in the tank and were characterized as “high activity”. Water was changed between each trial. A total of 27 to 31 individuals were tested from each CO_2_ level.

### Startle response experiment

**Fig. 1.**
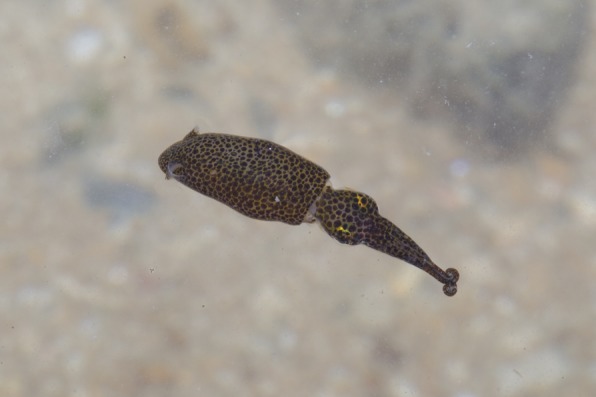
Photograph of pygmy squid. Photograph of squid employing defensive anchor posture and with darkened mottle display body pattern. Note posture and body pattern type can occur independently and result in additional combinations to those seen here. Photograph reproduced with the permission of the copyright holder, Ria Tan.

A looming visual shape was used to test the effect of elevated CO_2_ on the threat response of *I. pygmaeus*. This experiment was performed after the non-manipulative activity level observations. After the activity observations, each individual was transferred into a 17 L glass aquarium (34×20×25 cm). A computer monitor was set up against one side of the tank displaying an all-white screen while the remaining three sides of the tank were covered with white paper to both minimize external distractions and match the white screen of the computer monitor. Each squid was observed from behind a black plastic sheet for 90 seconds as an acclimatisation period. A startle response trigger was then remotely activated when the computer monitor appeared to be in the squid's field of vision and the squid was a maximum distance of 8.5 cm (or one fourth of the tank length) from the side containing the monitor with the startle stimulus. The startle response was a black oval shape, which expanded in size from zero to 15×18.7 cm in 1.15 seconds. The non-natural stimulus used in this experiment was used as a means to elicit a startle response from the squid, not necessarily to resemble a predator. A very similar visual startle stimulus from a computer monitor elicits a natural defensive response in damselfish (Fuiman et al., 2010). The reaction of each squid was recorded with a high-speed camera (Casio Exilim EX-FH20) positioned on a tripod directly above the aquarium (44 cm from lens to surface of water), at 300 fps. In analysing the video, we determined: time to react, type of reaction, distance moved, if ink was discharged and body pattern choice. Reaction types included a “jet” response, defined as propulsion or movement of a distance greater than 2 cm from the initial starting position in a direction away from the visual startle stimulus, and an “anchor posture” (Fig. 1), defined as holding position and orienting towards the threat with an upwards extended arm posture (Moynihan, 1983). Body pattern choices were categorised as “no change” (remaining in a darkened mottle pattern), “deimatic display” (darkened mottle pattern accompanied by larger dark spots or dark stripes along the side of the mantle) (Moynihan, 1983; Hanlon and Messenger, 1996), “transparent” (body colour change to transparent), and “flash” (squid produces a rapid succession of body patterns) (Moynihan, 1983). The size of the experimental tank is not expected to have had a limiting effect on squid responses to the visual stimulus as the distance moved by individuals was never more than half the length of the tank, and animals were not restricted in their escape behaviours as they did not jet into the walls of the aquarium. The mantle length of each squid was recorded with callipers after the startle experiment.

### Data analysis

Statistical analyses were performed using S-PLUS statistical software (TIBCO Software Inc., Spotfire S+ Version 8.2). Preliminary analyses showed that there were no significant differences among replicate treatment tanks for any of the traits measured; therefore data were pooled by CO_2_ treatment. A chi-square test of heterogeneity was used to test for independence between the activity levels of the squid at the three CO_2_ treatment levels. A negative binomial GLM was also performed to test if squid size had an effect on activity under the three CO_2_ treatments. The negative binomial was used to describe over-dispersed (zero-inflated) count data (variances > mean), rather than Poisson distribution data (variance  =  mean). Coefficients from the negative binomial correlation of coefficients table (t-values) were converted into p-values. A Wilcoxon signed rank-sum test was used to test for differences in movement (number of line crosses) among the three CO_2_ treatments for all animals in the experiments (including ‘at rest’ animals), as the data were not normally distributed. Analysis of variance (ANOVA) on log-transformed data was then used to test for differences in movement among CO_2_ treatments for active animals only.

Logistic regression (binomial distribution) and Fisher's Exact tests were used to test for variation in squid behaviours among the three CO_2_ treatments. The activity level of each individual from the observation study was included in the logistic regression as a continuous variable to determine if this correlated with their inking and reaction type in the startle response experiment. Logistic regression could not be used to compare body pattern choices among CO_2_ treatments, because there were more than 2 classes of behaviour in this analysis; therefore, a Fisher's Exact test was used for this comparison. These analyses are well suited to the relatively small sample sizes and zero-inflated data for these behavioural traits. One-way ANOVA with Tukey's HSD post-hoc test, with CO_2_ levels as a fixed factor, were used to determine the effect of CO_2_ treatments on squid reaction time and escape distance. Prior to the analysis of distance moved, we performed a Pearson's correlation to determine that the initial distance from the startle stimulus did not affect the subsequent escape distance moved (p = 0.356). Data for reaction time and escape distance met assumptions of normality and homogeneity of variances (Levene's test) confirming that the data were appropriate for ANOVA.

## RESULTS

### Activity observations

**Fig. 2.**
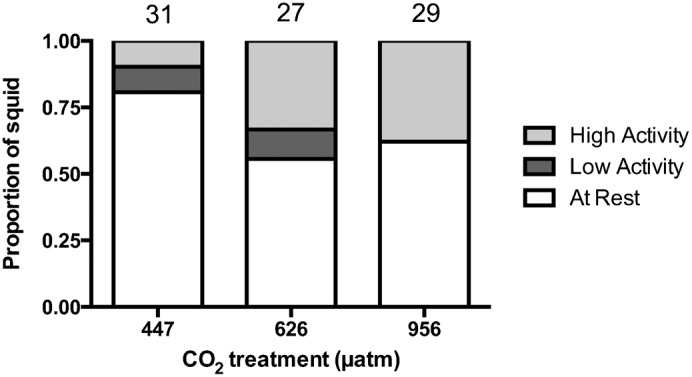
Effect of elevated CO_2_ on resting frequency of squid. Proportion of squid that were at rest (zero line crosses), displayed low activity levels (1–25 line crosses), and high activity levels (more than 25 line crosses) in behavioural trials for individuals exposed to control (447 µatm), moderate (626 µatm), or high (956 µatm) CO_2_. Sample sizes are displayed above the bars.

**Fig. 3.**
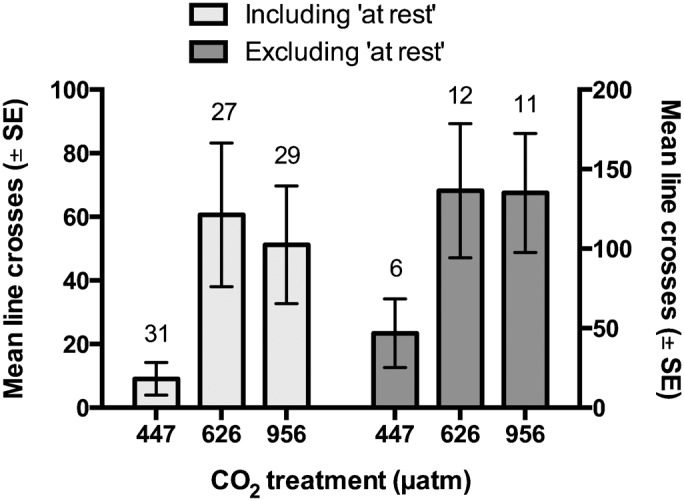
Effect of elevated CO_2_ on the movement levels of squid. Mean number of line crosses (± standard error) in 5-minute trials for squid exposed to control (447 µatm), moderate (626 µatm), or high (956 µatm) CO_2_. Mean movement includes individuals that were at rest throughout the trial (zero line crosses) in light grey (left *y*-axis) and excludes individuals at rest in dark grey (right *y*-axis). Sample sizes are displayed above the bars.

There was a significant difference in the frequency of squid at rest and active among control and elevated CO_2_ treatments (χ^2^ = 9.77, *df* = 4, p = 0.044) (Fig. 2). During the 5-minute observation period, 81% of squid from the control treatment remained at rest (attached to the aquarium wall), whereas only 56% of squid from the moderate CO_2_ treatment and 62% from the high CO_2_ treatment remained at rest (n = 27–29). Additionally, some squid from the control and moderate CO_2_ treatment, but not the high CO_2_ treatment, had a low level of activity (1–25 line crosses). Further analysis of movement levels showed control squid had a mean of 10 (SD = 28) line crosses compared to 61 (SD = 117) and 51 (SD = 100) line crosses in the moderate and high CO_2_ treatments, respectively, when all individuals (including those at rest) were included (Fig. 3). For all individuals (n = 27–29), the mean number of line crosses was significantly different between control and moderate CO_2_ treatments (Wilcoxon rank-sum test; Z = −2.272, p = 0.023), but not between the control and high CO_2_ treatments (Wilcoxon rank-sum test; Z = 1.898, p = 0.057). Among active individuals only (n = 6–12), there was a significant difference in the mean number of line crosses among the control and elevated CO_2_ treatments (ANOVA: F_2,26_ = 3.474, p = 0.046). A post hoc test showed that while there was no significant difference between control and moderate CO_2_ treatments (Tukey HSD; p = 0.141), there was a significant difference between the control and high CO_2_ treatment levels (Tukey HSD; p = 0.038). Activity levels were not affected by individual body size (negative binomial GLM; p = 0.635).

### Response to visual stimulus

In the startle response trials, there was no significant difference among treatments in the mean time to react to the looming stimulus (n = 10–16) (ANOVA: F_2,37_ = 1.442, p = 0.249). The mean reaction times among CO_2_ treatments were between 40 and 60 ms. Similarly, there was no significant difference among treatments in the distance moved in response to the stimulus (n = 19–23) (ANOVA: F_2,30_ = 0.135, p = 0.874), which ranged from 8.7 to 9.9 cm.

**Fig. 4.**
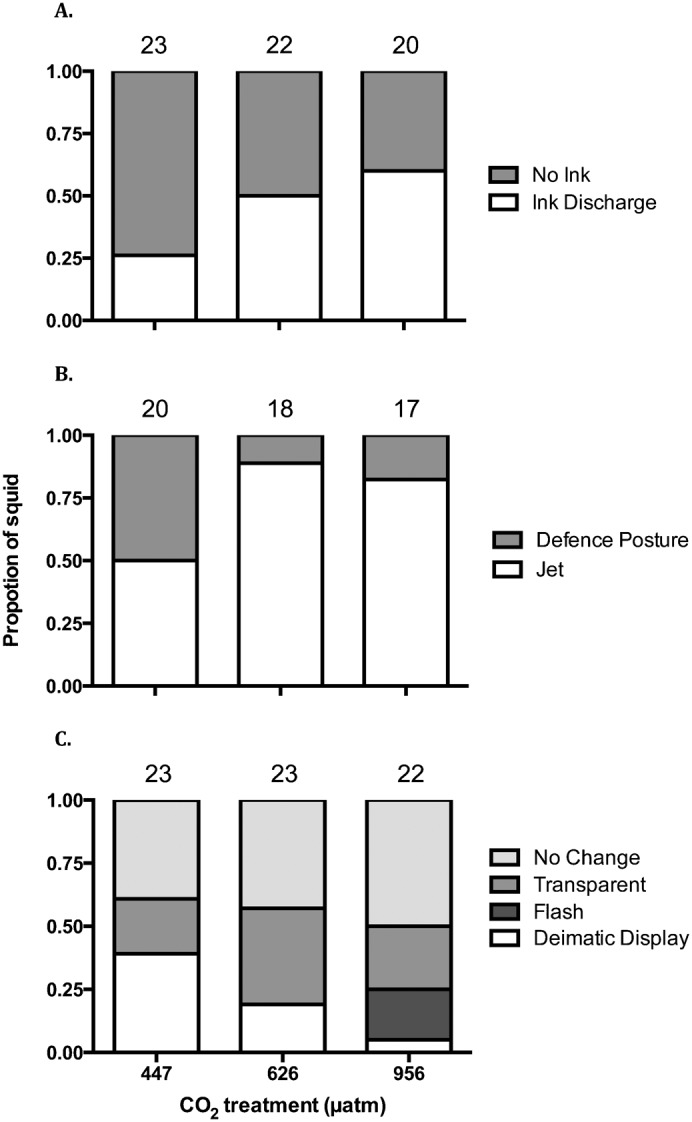
Effect of elevated CO_2_ on squid response to startle stimulus. Proportion of squid that (a) produced ink as a defensive mechanism, (b) chose a defensive “anchor” posture or jet response, (c) displayed various body patterns in response to a visual startle stimulus from three CO_2_ treatment levels: control (447 µatm), moderate (626 µatm), or high (956 µatm). Sample sizes are displayed above the bars.

There was a non-significant trend towards an increased frequency of squid discharging ink in response to the looming stimulus increased at higher CO_2_ levels (Fig. 4A; Logistic Regression: χ^2^ = 5.399, *df* = 2, p = 0.067). At control levels, only 26% of squid discharged ink when startled, whereas 50% at moderate and 60% at high CO_2_ treatments discharged ink (n = 20–23). When ink discharge was further compared by pairwise Fisher's Exact tests, there was a significant difference in the frequency of squid discharging ink between the control and high CO_2_ group (p = 0.033), but not between the control and moderate CO_2_ group (p = 0.129). There was no correlation between the observed activity level and the tendency to discharge ink in response to the visual startle stimulus (Logistic regression: χ^2^ = 0.136, *df* = 1, p = 0.712).

Behavioural responses to the looming stimulus were separated into two reaction type categories: jet and defensive anchor posture. There was a significant difference in reaction type among the three treatments (n = 17–20) (Fig. 4B; Logistic regression: χ^2^ = 9.863, *df = 2*, p = 0.007). Controls had a 50% split between these two reaction types, whereas at higher CO_2_ levels, a greater proportion of animals jetted away from the stimulus (89% at moderate CO_2_, 82% at high CO_2_). There was also no correlation between the observed activity level and the behavioural response to the visual startle stimulus (Logistic regression: χ^2^ = 0.114, *df* = 1, p = 0.734).

Body patterns displayed were significantly different among CO_2_ levels (n = 22–23) (Fisher's exact test; p = 0.024). The difference was most notable between the control and high CO_2_ treatments (Fig. 4C) where there was a sharp decline in the proportion of squid that utilized the deimatic display body pattern from 39% to 5%. Lastly, the flashing display was only observed in the high CO_2_ treatment, with 20% of the squid displaying this pattern.

## DISCUSSION

CO_2_ concentrations projected to occur in the oceans by the end of the century may affect some behaviours of male pygmy squid *Idiosepius pygmaeus* in an aquarium setting. Elevated CO_2_ increased the number of individuals that were active, as opposed to resting on the side of the tank, as well as increased general levels of movement among individuals that were active. In response to a visual stimulus, there was no difference in the time taken to react or in the distance moved. However, the type of reaction shifted towards the jet escape response rather than anchor posture as CO_2_ increased and the proportion of squid inking increased at the highest CO_2_ treatment. Additionally, elevated CO_2_ was linked to a change in body pattern choice. Consequently, it appeared that squid retained the physical ability to escape at elevated CO_2_, however, their behavioural choices were altered, as previously observed in fishes (Domenici et al., 2012; Nilsson et al., 2012) and other molluscs (Manríquez et al., 2014; Watson et al., 2014).

The number of squid that were active, as opposed to remaining at rest by use of their adhesive gland, was greater in the elevated CO_2_ treatments. Although the proportion of active squid was highest in the moderate CO_2_ treatment, there were no squid in the ‘low activity’ category in the high CO_2_ treatment. Squid in the ‘low activity’ category were predominately floating at the surface using deceptive camouflage while making minor movements with their fins, as opposed to the squid in the ‘high activity’ category which were actively jetting throughout the tank. Therefore, while these ‘low activity’ squid did have a number of line crosses, they displayed a different type of behaviour than those that were highly active. Like most cephalopods, pygmy squid rely on crypsis and deceptive camouflage to avoid detection by predators (Moynihan, 1983). Individuals that remained at rest during the activity level trial changed their skin tone to pale or transparent while attached to the aquarium wall, becoming well camouflaged. Squid are soft-bodied and vulnerable, so employing cryptic behaviours as a primary defence is imperative to their survival (Semmens et al., 1995; Hanlon and Messenger, 1996). Additionally, immobility is crucial to effective crypsis as most predators are sensitive to motion in their visual fields (Curio, 1976). Spending less time deploying cryptic behaviours at elevated CO_2_ may potentially place pygmy squid at greater risk of being detected by a predator.

Pygmy squid also exhibited higher levels of general movement at elevated CO_2_ by completing a higher number of line crosses during the observation period. Among active animals, squid in the elevated CO_2_ treatments had a near 3-fold increase in movement compared with controls. Therefore, as well as reducing the number of squid that used their adhesive glands to remain at rest, elevated CO_2_ increased the levels of movement in those individuals that were active. Squid have finely balanced energy budgets (Rodhouse, 1998) and increased movement could affect individual energy budgets. If increased activity extended to wild populations, it may have adverse effects on defensive options. For example, increased movement during other active behaviours, such as courtship and foraging causes a decrease in vigilance in some cephalopods (Mather, 2010). In contrast to the increased activity in pygmy squid at elevated CO_2_ levels in this study, Rosa and Seibel observed a 45% decrease in activity levels in the jumbo squid (*Dosidicus gigas*) at elevated CO_2_ (1000 µatm) (Rosa and Seibel, 2008). This may indicate different responses among species, however, jumbo squid activity levels were measured by rates of mantle contraction for jet propulsion in a constricted chamber, therefore this measure of activity may not be directly comparable to the less restricted line-cross activity observations made here.

Along with alterations to activity, elevated CO_2_ caused changes in the secondary defensive behaviours. While time to react and distance moved in response to the visual startle stimulus was not affected by elevated CO_2_, squid exhibited very different response types and defensive behaviours. In the elevated CO_2_ treatments, a much higher proportion of squid jetted instead of displaying the defensive anchor posture compared to controls. This demonstrates a shift from secondary defence behaviours towards escape responses at elevated CO_2_. Longfin squid (*Loligo pealeii*) alter their defensive tactics depending on the type of predator encountered (Staudinger et al., 2011), indicating that squid choose from various defensive options depending on the situation. However, in our experiment, the different behaviours were observed among CO_2_ treatment levels rather than the type of threat encountered.

Squid were more than twice as likely to discharge ink at the highest CO_2_ level compared to controls. Across all treatments, in all but one instance, squid released ink in the ‘blanch-ink-jet’ manoeuvre, an escape behaviour, which involves blanching body colours and inking as the animal jets away (Hanlon and Messenger, 1996). When ink is discharged during this manoeuvre, it is released as a ‘pseudomorph’, which is a collection of ink held together by mucus in about the same size and shape as the squid. This serves to hold the visual attention of the predator while the squid escapes. In the one isolated instance in which a squid released ink in an alternative method, it discharged ink in a large cloud known as a ‘smoke screen’ and did not jet; this however is still described as an escape behaviour (Hanlon and Messenger, 1996). Inking behaviour is used as a defence in response to only the most threatening situations (Moynihan and Rodaniche, 1982), suggesting that squid reserve inking as a last line of defence. This could be because a discharge of ink would potentially give away the presence of the squid if it had not already been detected, so in some situations it may be the safer decision to choose not to ink. The energetic cost of producing ink has been little studied, but it is not cost free, and squid may also reserve this behaviour as a last resort as a means of conserving energy. The finely balanced energy budgets of squid (Rodhouse, 1998) may be affected by increased inking as this could reduce the energy available to other activities, such as maintenance and reproduction.

Lastly, there was a significant difference among CO_2_ treatment levels in squid body pattern choice. Notably, squid at the high CO_2_ treatment were the only ones to display the flash of body patterns. There was also a strong negative trend in the proportion of animals that responded with the deimatic display with increasing CO_2_ levels. The deimatic display is a secondary defence response used to threaten or bluff the predator while a flash of displays is an escape behaviour used only after an attack is imminent and aims to confuse or disorient the predator in order to provide a window of escape (Humphries and Driver, 1970; Edmunds, 1974; Moynihan, 1983; Hanlon and Messenger, 1996). Our results suggest that squid may avoid secondary defence responses and instead respond with escape behaviours as CO_2_ levels increase. It is important to note that there was no correlation between the performance of individuals in the activity level observations and their behavioural response to the startle stimulus experiment. This indicates that the activity levels of individuals observed did not influence the behavioural responses seen in the startle stimulus trial.

The results of this study are discussed for male pygmy squid. The number of available females compared to males was too low to investigate any sex-specific effects. The sex ratio of this species has not been determined (Boletzky et al., 2005), but the lack of females found could be representative of natural sex-ratios or could be due to the potential solitary nature of females (Lewis and Choat, 1993). Further work is needed to determine if the behavioural changes reported here are sex-specific or, potentially, more broadly applicable to populations of this species. This study indicates that CO_2_ levels in the oceans projected for the end of this century may alter some behaviours in male pygmy squid including defensive responses. However, the mechanism behind any behavioural changes (such as altered neural function or metabolism) in *I. pygmaeus* as a result of elevated CO_2_ is not yet known and further research is required. In fishes, behavioural alteration occurs due to interference with the function of GABA_A_ neurotransmitter receptors (Nilsson et al., 2012; Hamilton et al., 2014; Chivers et al., 2014). Although squid possess GABA_A_ receptors, they are thought to primarily use L-glutamate transmitters and AMPA/kainite ionotropic receptors (Di Cosmo et al., 2006). Nevertheless, elevated CO_2_ potentially interferes with GABA_A_-like receptor function in other marine molluscs (Watson et al., 2014) and indicates a potential for similar effects in squid. Furthermore, the bigfin reef squid (*Sepioteuthis lessoniana*) shows a stabilization of extracellular pH accompanied by an increase in blood HCO_3_^−^ levels after prolonged exposure to elevated CO_2_ (Hu et al., 2014), which is similar to the response seen in fish for controlling acid–base balance to regain optimal GABA_A_ function (Nilsson et al., 2012). Investigating the mechanism of behavioural alteration was outside the scope of this study and these hypotheses would need to be examined in future research.

This study is the first to demonstrate the potential for near-future CO_2_ levels to alter behaviours in a cephalopod and may alter the behaviours in ways that could increase their risk of predation. However, whether this shift from secondary defence to escape responses is an adverse effect, or if it would actually increase survival in the wild, is not yet clear. Minor injury in the squid *Doryteuthis pealei* leads to an increase in responsiveness to threats resulting in higher survival rates (Crook et al., 2014). Therefore, it is possible that exposure to elevated CO_2_ could cause a sensitized state in pygmy squid, during which squid choose escape responses as a potentially more successful option. Also, squid inhabit a wide range of environmental conditions and have varying behaviours and physiologies, therefore it is unknown whether these results will extend to other species. There is evidence that some cephalopods have the ability to cope with elevated CO_2_ in terms of growth and calcification (Gutowska et al., 2008; Gutowska et al., 2010) and acid–base regulation (Hu et al., 2013; Hu et al., 2014). The extremely fast growth rates of squid and the rapid rates of turnover at the population level indicate that they have potential to adapt quickly to environmental change over generations (Gutowska et al., 2010). Therefore, it is possible that *I. pygmaeus* might have considerable capacity to adapt their behavioural responses during the time frames (next 35–85 years) that the levels of ocean acidification tested here will occur (Munday et al., 2013a; Sunday et al., 2014). Due to their very short lifespans (∼90 days), pygmy squid could be an ideal model species for future studies to test the potential of transgenerational acclimation and adaptation to ocean acidification in metazoans.

Our results suggest that elevated CO_2_ can alter activity and defensive behaviours in a tropical squid. Squid are important in ecosystems as both predator and prey, and any impacts of ocean acidification on squid may affect marine ecosystems. As more studies are conducted, it is becoming apparent that rising CO_2_ levels in the ocean could affect the behaviours of a wide range of marine species. Determining the extent of these effects as well as assessing the potential for adaptation will be necessary to properly predict the effects of ocean acidification on marine ecosystems.
